# Clinical and genetic factors associated with anxiety and depression in breast cancer patients: a cross-sectional study

**DOI:** 10.1186/s12885-021-08615-9

**Published:** 2021-07-30

**Authors:** Aline HAJJ, Roula HACHEM, Rita KHOURY, Souheil HALLIT, Bashar ElJEBBAWI, Fady NASR, Fadi EL KARAK, Georges CHAHINE, Joseph KATTAN, Lydia RABBAA KHABBAZ

**Affiliations:** 1 Faculty of Pharmacy, Saint-Joseph University, Beirut, Lebanon; 2 Laboratoire de Pharmacologie, Pharmacie Clinique et Contrôle de Qualité des Médicaments, Faculté de pharmacie, Saint-Joseph University, Beirut, Lebanon; 3 Faculty of Medicine and Medical Sciences, Holy Spirit University of Kaslik (USEK), Jounieh, Lebanon; 4 INSPECT-LB (Institut National de Santé Publique d’Épidémiologie Clinique et de Toxicologie-Liban), Beirut, Lebanon; 5 Research Department, Psychiatric Hospital of the Cross, Jal Eddib, Lebanon; 6 Department of Hemato-Oncology, Hôtel-Dieu de France Hospital, Faculty of Medicine, Saint-Joseph University of Beirut, Beirut, Lebanon

**Keywords:** Anxiety, Breast cancer, *COMT*, Depression, HADS, *PER2*, Pharmacogenetics

## Abstract

**Background:**

Despite the progress in assessment and treatment of breast cancer, being diagnosed with it or receiving chemotherapy treatment is still conceived as a traumatic experience. Women develop negative thoughts about life and death with detrimental effects on their daily physical functioning/activities, emotional state and overall quality of life. The aim of our study was to evaluate the level of anxiety and depression among breast cancer patients receiving chemotherapy and explore the correlation between these psychological disorders, clinical, sociodemographic and genetic factors.

**Methods:**

A cross-sectional study was conducted among breast cancer patients undergoing intravenous chemotherapy at the oncology outpatient unit of Hôtel-Dieu de France hospital (November 2017–June 2019; Ethical approval number: CEHDF1016). All patients gave their written informed consent and completed several validated scales, including the Hospital Anxiety and Depression scale (HADS) for the assessment of anxiety and depression. Sleep quality, insomnia, cognitive function, fatigue and pain were also evaluated. Genotyping for certain gene polymorphisms (*CLOCK, PER2, CRY2, OPRM1, ABCB1*, *COMT, DRD2*) was performed using the Lightcycler® (Roche).

**Results:**

A total of 112 women was included. The prevalence of depression was 43.4%, and 56.2% of the patients reported anxiety (based on the HADS classification). Multivariable analysis showed that higher cognitive scores and taking fosaprepitant were significantly associated with lower depression and anxiety scores. Moreover, being married compared to single was also associated with lower depression scores, whereas higher PSQI scores (worse sleep quality) and having the *PER2* AA variant genotype compared to GG were significantly associated with higher depression scores. Finally, reporting a more severe insomnia and having the *COMT* Met/Met genotype were significantly associated with a higher anxiety score.

**Conclusions:**

Our study demonstrated a strong relationship between depression scores and cognitive impairment, sleep quality, marital status, fosaprepitant intake, and *PER2* polymorphism, while anxiety scores were correlated to cognitive impairment, insomnia severity, fosaprepitant intake, and *COMT* polymorphism. The association with *PER* polymorphism was not previously reported. Identification of genetic and clinical risk factors for anxiety and depression would help clinicians implement an individualized management therapy aiming at preventing and alleviating the burden of these symptoms in breast cancer patients, hence improving their overall quality of life.

## Introduction

Despite the improvement of survival rates due to the progress in assessment and treatment of breast cancer, being diagnosed with it or receiving chemotherapy treatment is still conceived as a traumatic experience. Women develop negative thoughts about life and death with detrimental effects on their daily physical functioning/activities, emotional state and overall quality of life [1, 2]. Patients find difficulties coping with these challenges [3, 4] and previous research has estimated the prevalence of anxiety and depression in breast cancer patients from 13 to 54% [5]. One of the major concerns among these patients is the fear from cancer progression and its recurrence, thus increasing susceptibility to anxiety and depressive disorders [6].

Several factors can contribute to the psychological distress; evidence suggests that identifying and assessing these clinical/genetic biomarkers would provide greater insight into depression and anxiety in breast cancer patients under treatment [7, 8]. Cancer treatments such as mastectomy and chemotherapy can lead to changes in self-concept: physical changes (scarring, hair loss, weight gain, etc.), lower self-esteem and self-efficacy, all leading to alterations in the emotional well-being. Sociodemographic factors could also be responsible, including age [9–11], marital status [12], educational level and financial income [13]: young single women with lower educational level and lower financial support tend to exhibit higher levels of anxiety and depression among breast cancer patients [14–16].

As for genetic factors implicated in anxiety and depression most of the studies focused on the genes of the serotonin pathways [8, 17–19] and pro-inflammatory cytokines [8, 20]. Additional neuronal circuits may be potentially involved in mood regulation, such as circadian rhythm dysregulations [21, 22] (exploration of genes of the circadian rhythm), stress response disruptions via the hypothalamic-pituitary-adrenocortical (HPA) axis activity (implicating the opioid system along with drug efflux transporters at the blood brain barrier -BBB- regulating cortisol access into the central nervous system), and dopamine neurotransmission impairments [23, 24]. Studies investigating the relationship between these circuits and anxiety/depression among cancer patients are scarce, hence the choice of selected single nucleotide polymorphisms (SNPs) in genes involved in the these pathways.

Three genes in the circadian rhythm regulation have been explored in our study: the core member *Circadian Locomotor Output Cycles Kaput CLOCK* gene (SNP c.3111T>C; rs1801260), the *Period 2* (*PER2*) *gene* (rs934945; G>A) and the *Cryptochrome circadian Regulator 2* (*CRY2*) gene (rs10838524; G>A). Very few studies explored their implication in mood disorders (*CRY2* SNP being associated with persistent depressive disorder [25]) but none of them in breast cancer patients.

Furthermore, we decided to study the SNP c.118A>G (rs1799971) of *OPRM1* that encodes the mu-opioid receptor. In fact, the opioid system has been shown to be involved in emotion regulation, HPA axis activity and stress responses [26, 27]. No previous studies were performed in breast cancer patients; however, in other populations, Slavich et al. have demonstrated an association between this polymorphism and depressive symptoms and patients with at least one G variant allele exhibited more depression symptoms compared to AA patients [28]. Other authors demonstrated an alteration in the endogenous opioid neurotransmission: the binding potential of mu-opioid receptors being significantly lower in women with major depressive disorder relatively to nondepressed women, which correlates to their negative affect ratings [29].

In the same context of HPA axis, some studies suggested that transmembrane efflux transporters, such as P-glycoprotein (P-gp), might affect the susceptibility of patients to HPA over-activity, commonly described in depression [30]. Fujii et al. reported, in a case-control study, that the mutant TT genotype for the rs1045642 (c.3435T>C, the one selected in our study) in *ABCB1*, the gene encoding P-gp, was significantly more common in patients with major depressive disorder than in controls [31]. Authors suggested that TT patients might efflux less cortisol out of the central nervous system predisposing them to depression.

Finally, regarding the disruption in dopaminergic neurotransmission, we evaluated the SNP p.Val158Met (rs4680) of *COMT* gene encoding the catechol-O-methyltransferase (COMT) that plays a key role in the metabolism of catecholamines particularly adrenaline and noradrenaline, also known as “stress hormones”, and dopamine [32]. The SNP c.957C>T (rs6277) in the dopamine receptor 2 gene (*DRD2*) was also selected for evaluation since it has been significantly associated with anxiety in alcohol-dependent patients [33].

Correctly assessing these symptoms as well as the associated factors is essential in order to improve the quality of life of patients. Therefore, we conducted this cross-sectional study to: i. explore the prevalence and severity of anxiety and depression in a group of breast cancer patients undergoing intravenous chemotherapy course; ii. examine the relationship between these two mood disorders and sociodemographic, clinical and genetic factors.

## Subjects and methods

### Study design

We conducted a cross-sectional study, from November 2017 till June 2019, at Hôtel-Dieu de France (HDF) University Hospital exploring the anxiety and depression of women with primary breast cancer treated by chemotherapy.

### Ethical approval

The hospital ethical committee approved the study (Reference: CEHDF1016, July 2017) and all patients signed a written consent prior to inclusion. All methods were carried out in accordance with relevant guidelines and regulations.

### Patient’s sociodemographic and clinical information

All women aged at least 18 years old, diagnosed with a breast cancer and scheduled to receive an intravenous chemotherapy regimen every 21 days (random cycle out of a maximum of 10 cycles) at the outpatient oncology unit at HDF, were included in the study. Non-inclusion criteria consisted of relapse/other types of cancer, concomitant radiation therapy, brain metastasis or any medical/surgical disorder of the central nervous system (dementia, multiple sclerosis, epilepsy, Parkinson’s disease, mental retardation and neurosurgeries). None of the patients was receiving adjuvant hormone therapy at the time of the evaluation [34–36].

Two trained pharmacists collected demographic and clinical information related to the patient (medical records or interview with patients): age, gender, body mass index (BMI; based on the patient’s weight -measured the day of the admission to HDF for treatment- and height), Body Surface Area (BSA as calculated by the Mosteller formula) [37, 38], ethnicity/nationality, marital status, education level, socioeconomic level, presence of comorbidities (diabetes, hypertension, dyslipidemia, others) and current alcohol consumption (self-declared by the patient), current tobacco smoking (self-declared by the patient), and medications (other than chemotherapy). Data related to cancer were also noted: type of cancer, metastases, number of chemotherapy cycles, chemotherapy regimen (chemotherapy agents and doses/m^2^). The pharmacists also assisted patients in completing the self-reported questionnaire, evaluating anxiety and depression along with other disorders (sleep, cognition, fatigue, and pain), and ensured that all questions were answered (day 1 of chemotherapy, random cycle). The number of cycles was recorded as the “number of chemotherapy cycles”. More details are described elsewhere [39].

### Clinical assessments

Patients filled the questionnaires the day they were admitted to the oncology unit to take their chemotherapy regimen (day 1 of chemotherapy, random cycle). The number of the cycle was then recorded as “number of chemotherapy cycle”.

#### Anxiety and depression

The Hospital Anxiety and Depression Scale (HADS) was used to self-assess anxiety and depression. It consists of two subscales designed to identify and quantify anxiety (HADS-A) and depression (HADS-D) in patients. Symptoms reported during the previous week are reported on a scale from “0” (not at all) to “3” (most of the time). Patients would be classified as “normal” (Score 0–7), having a “borderline anxiety/depression” (Score 8–10), or “Clinical anxiety/depression” (Score 11–21) [40].

#### Cognitive function assessment

The Functional Assessment of Cancer Therapy-Cognitive Function (FACT-Cog, version 3; Licensing agreement granted on November 2, 2017) was used to self-assess cognitive functioning. The scale consisted of 37 questions, allowing the evaluation of four different domains: 1. Perceived cognitive impairments subscale (CogPCI; 20 items); 2. Perceived cognitive abilities subscale (CogPCA; 9 items); 3. Comments from others subscale (CogOth; 4 items); and 4. Impact of perceived cognitive impairments on quality of life subscale (CogQOL; 4 items). Higher total FACT-Cog score/subscore indicates better cognitive function and lower impact on the quality of life of patients [41].

#### Sleep assessment

Two tools were used for sleep assessment: 1. the Pittsburgh Sleep Quality Index (PSQI; 19 questions related to the last month), designed to measure 7 domains: subjective quality of sleep, sleep latency, sleep duration, sleep efficiency, sleep disorders, sleep medication and daytime dysfunction. The component scores range from 0 (no difficulty) to 3 (severe difficulty) and allows the calculation of an overall score ranging from 0 to 21 [42]; and 2. The insomnia severity index (ISI; 7 items), allowing the self-assessment of the severity of insomnia during the past two weeks. The measurement on a scale of 5 points ranging from 0 (very satisfied) to 4 (not at all satisfied). Total scores range from 0 to 28, higher scores representing a more severe insomnia [43].

#### Fatigue and pain assessment

The EORTC-QLQ C30 scale (European Organization for Research and Treatment of Cancer Quality of Life Questionnaire) was used for the evaluation of fatigue using three questions: “Do you need rest?” (QLQ C10); “Did you feel weak?” (QLQ C12) and “Were you tired?” (QLQ C18) [44]. Pain was estimated by the visual analogue scale (VAS) ranging from 0 (absence of pain) to 10 (maximum of pain) [45].

### DNA sampling and genotyping

DNA was obtained using a buccal swab (Whatman® FTA® card technology-GE Healthcare) as recommended by the manufacturer. Genotyping for the studied SNPs was performed using the Lightcycler® 2.0 (Roche Diagnostics GmbH-Mannheim-Germany): *CLOCK* (rs1801260), *PER2* (rs934945), *CRY2* (rs10838524), *OPRM1* (rs1799971), *ABCB1* (rs1045642), *COMT* (rs4680), and *DRD2* (rs6277). Positive (defined by direct sequencing) and negative controls (water) were systematically included in experiments. Details regarding the PCR protocol and conditions are presented in Supplementary material 1.

### Data and statistical analysis

The SPSS software version 25.0 was used for statistical calculations. Descriptive statistics were calculated for all variables in the study. This includes the mean and standard deviation for continuous measures, counts, and percentages for categorical variables. Deviation from the Hardy-Weinberg equilibrium was tested using χ^2^ analysis with one degree of freedom. Since the sample consisted of more than 100 women [46], parametric tests were used. The normality of distribution of the depression and anxiety scores were checked via: 1- a visual checking of the normality that showed a near normal distribution; in all cases, whenever a sample size is > 30–40, a departure from normality does not affect the results in a major way, and parametric tests can be used” [47]; 2- A calculation of the skewness and kurtosis; values for asymmetry and kurtosis between − 1 and + 1 are considered acceptable in order to prove normal univariate distribution [47] (For the anxiety score: skewness = 0.301; kurtosis = − 0.824; for the depression score: skewness = 0.238; kurtosis = − 0.905). Parametric tests included the Student t test to compare two means, ANOVA to compare three or more means, and Pearson correlation test to correlate two continuous variables. Forward linear regressions were performed taking anxiety and depression as dependent variables. Alleles of genotypes were one time taken as three categories and another combined together and checked for any significant association with both anxiety and depression. Variables that showed *p* < 0.2 in the bivariate analysis were taken as independent variables in the multivariable analyses to eliminate potential confounding factors as much as possible. A value of *p* ≤ 0.05 was considered statistically significant.

## Results

### Patients demographic and clinical characteristics

Our study included 112 breast cancer women (mean age 56.04 ± 11.69 years with an average BSA of 1.75 ± 0.16 m^2^). The average number of chemotherapy cycles was 4.45 ± 2.35 cycles. Among these patients, only 8 had metastasis and were treated by a palliative chemotherapy regimen (Table 1). The mean HADS-A score was 8.69 ± 5.25, with more than half of the patients self-reporting clinical anxiety (19.6% borderline anxiety and 36.6% anxiety). As for depression, the mean HADS-D score was 7.27 ± 4.59 with 43.4% of patients reporting depression. More details are presented supplementary material 2.

**Table 1:** Patients’ demographics and clinical characteristics (*N* = 112)*

		Frequency (%)
Marital status	Single	15 (13.4%)
	Married	94 (83.9%)
	Widowed	3 (2.7%)
Level of education*	Primary	16 (14.5%)
	Secondary	61 (55.5%)
	University	33 (30%)
Profession/Work	No	78 (69.6%)
	Yes	34 (30.4%)
Socioeconomic status	Low	8 (8%)
	Middle	96 (85.7%)
	High	7 (6.3%)
Alcohol consumption	Yes	18 (16.1%)
Smoking	Yes	26 (23.2%)
	Previous smoker	4 (3.6%)
Hypertension	Yes	30 (26.8%)
Diabetes	Yes	11 (9.8%)
Dyslipidemia	Yes	23 (20.5%)
Presence of metastases	No	104 (92.9%)
	Yes	8 (7.1%)
Type of metastases	Bone	6 (75%)
	Lung	2 (25%)
Type of chemotherapy	Adjuvant	79 (71.2%)
	Neoadjuvant	24 (21.6%)
	Palliative	8 (7.2%)
	**Mean ± Standard Deviation (SD)**	**Median [25–75 Percentiles]** ^‡^
Age (years)	56.04 ± 11.69	56 [49–65]
Body Mass Index (BMI; Kg/m^2^)	25.90 ± 4.62	25.65 [23.46–28.14]
Body Surface Area (BSA; m^2^)	1.75 ± 0.16	1.74 [1.66–1.86]
Number of chemotherapy cycles	4.45 ± 2.35	4 [2–6]
Pain VAS score	1.68 ± 2.49	0 [0–3]
**Psychological factors**		
HADS-A	8.69 ± 5.25	9 [4–13]
*Normal (n; %)*	*49 (43.8%)*	
*Borderline anxiety (n; %)*	*22 (19.6%)*	
*Anxiety (n; %)*	*41 (36.6%)*	
HADS-D	7.27 ± 4.59	7 [3–11]
*Normal (n; %)*	*60 (53.6%)*	
*Borderline depression (n; %)*	*22 (16.6%)*	
*Depression (n; %)*	*30 (26.8%)*	
**Sleep evaluation**		
Insomnia Severity Index (ISI) score	10.44 ± 7.19	9 [5–15.75]
Pittsburgh Sleep Quality Index (PSQI) score	8.91 ± 4.63	9 [5–12]
**Cognition~**		
CogPCI score	56.94 ± 14.29	58.5 [49–67]
CogPCA score	22.99 ± 5.55	24 [20–26]
CogOth score	13.28 ± 3.17	14 [12–16]
CogQOL score	10.02 ± 4.79	10 [6–14.75]
Total FACT-Cog score	103.25 ± 23.15	107 [95–119]
**Fatigue Score**	42.12 ± 32.10	33.33 [11.11–66.67]

* Some variables did not sum up to 112 due to missing data

‡ Median and interquartile range were displayed since the variables’ distribution was not normal

~ CogPCI: Perceived Cognitive Impairments subscale; CogPCA: Perceived Cognitive Abilities subscale; CogOth: Comments from Others subscale; CogQOL: Impact of perceived cognitive impairments on Quality Of Life subscale

### Bivariate analyses

Bivariate analyses taking HADS-A or HADS-D scores as dependent variables showed that the intake of fosaprepitant (compared to not) and having the *OPRM1* AG genotype compared to AA were significantly associated with lower depression. The intake of fosaprepitant was also significantly associated with lower anxiety scores (Table 2). For genetic factors, bivariate analyses taking genotypes in two categories was also performed (Supplementary material 3); *CLOCK* SNP was the only factor that was significantly associated with depression: women with CC genotype had a significantly higher mean depression score compared to (TT and TC) patients.

**Table 2:** Bivariate analyses of categorical variables associated with anxiety and depression

Variable	Anxiety (HADS-A total score)	Depression (HADS-D total score)
**Education**		
Primary	8.37 ± 6.07	7.69 ± 5.40
Secondary	8.19 ± 5.36	6.87 ± 4.64
University	10.12 ± 4.46	8.03 ± 4.15
*p*	0.131	0.415
**Marital status**		
Single/widowed/divorced	9.89 ± 4.99	8.83 ± 4.34
Married	8.47 ± 5.30	6.98 ± 4.61
*p*	0.248	0.109
**Dyslipidemia**		
No	8.54 ± 5.31	7.09 ± 4.55
Yes	9.30 ± 5.11	8.00 ± 4.78
*p*	0.502	0.427
**Hypertension**		
No	8.58 ± 5.00	7.07 ± 4.53
Yes	9.00 ± 5.99	7.83 ± 4.82
*p*	0.885	0.437
**Cyclophosphamide intake**		
No	7.79 ± 5.08	7.30 ± 4.87
Yes	9.51 ± 5.32	7.25 ± 4.38
*p*	0.07	0.974
**Carboplatin intake**		
No	9.01 ± 5.23	7.57 ± 4.61
Yes	6.08 ± 4.89	4.83 ± 3.76
*p*	0.06	0.063
**Fosaprepitant intake**		
No	9.78 ± 5.51	8.03 ± 4.64
Yes	6.46 ± 3.97	5.67 ± 4.15
*p*	**0.002**	**0.014**
***CLOCK*** **rs1801260**		
TT	9.25 ± 5.38	7.25 ± 4.78
TC	8.13 ± 5.14	7.02 ± 4.48
CC	11.66 ± 2.51	9.33 ± 1.15
*p*	0.367	0.648
***PER*2 rs934945**		
GG	8.72 ± 5.61	6.86 ± 4.95
GA	8.56 ± 4.77	7.64 ± 3.85
AA	7.75 ± 3.20	9.00 ± 4.69
*p*	0.982	0.329
***CRY2*** **rs10838524**		
GG	8.54 ± 4.53	6.88 ± 3.93
AG	9.20 ± 5.56	7.50 ± 4.82
AA	7.17 ± 5.93	7.23 ± 5.50
*p*	0.343	0.865
***OPRM1*** **rs179971**		
AA	8.96 ± 5.26	7.77 ± 4.60
AG	7.35 ± 5.03	5.08 ± 3.87
*p*	0.170	**0.014**
***ABCB1*** **rs1045642**		
CC	9.05 ± 4.67	6.47 ± 3.96
CT	8.17 ± 4.99	7.43 ± 5.11
TT	8.68 ± 5.80	7.20 ± 4.41
*p*	0.760	0.789
***COMT*** **rs4680**		
Val/Val	8.63 ± 5.09	6.94 ± 4.45
Val/Met	7.84 ± 5.38	6.78 ± 4.33
Met/Met	10.12 ± 5.08	8.54 ± 5.25
*p*	0.168	0.362
***DRD2*** **rs6277**		
CC	10.29 ± 4.83	8.11 ± 4.73
CT	7.73 ± 4.84	6.63 ± 4.40
TT	7.97 ± 5.38	6.50 ± 4.59
*p*	0.218	0.406

Numbers in bold indicate significant *p*-values

Furthermore, higher CogPCI, CogOTH, CogQOL and total FACT-Cog scores were significantly associated with lower depression and anxiety scores, whereas a worse sleep quality (higher PSQI scores) and more severe insomnia (higher ISI scores) were significantly associated with higher depression and anxiety scores (Table 3).

**Table 3:** Bivariate analysis of continuous variables associated with anxiety and depression

Variable~	Anxiety (HADS-A)	***p***	Depression (HADS-D)	***p***
CogPCI	−0.387	**< 0.001**	− 0.376	**< 0.001**
CogPCA	−0.142	0.136	− 0.085	0.375
CogOTH	−0.340	**< 0.001**	−0.380	**< 0.001**
CogQOL	−0.263	**0.005**	−0.383	**< 0.001**
Total FACT-Cog	−0.374	**< 0.001**	−0.384	**< 0.001**
ISI	0.378	**< 0.001**	0.379	**< 0.001**
PSQI	0.355	**< 0.001**	0.370	**< 0.001**
Age	0.03	0.752	0.122	0.198
Body Mass Index (BMI)	0.016	0.866	0.079	0.410
Cycle number	−0.046	0.648	0.132	0.166
EVA	0.094	0.325	0.076	0.423
Fatigue score	0.122	0.327	0.125	0.312

Numbers in bold indicate significant *p*-values; CogPCI: Perceived Cognitive Impairments subscale; CogPCA: Perceived Cognitive Abilities subscale; CogOth: Comments from Others subscale; CogQOL: Impact of perceived cognitive impairments on Quality Of Life subscale

#### Multivariable analysis taking the total FACT-cog score as an independent variable

The results of a forward linear regression, taking the depression score as the dependent variable, showed that higher total FACT-Cog (B = -0.06), and the intake of fosaprepitant compared to not (B = -1.82) were significantly associated with lower depression scores, whereas higher PSQI scores (worse sleep quality) (B = 0.30) was significantly associated with higher depression scores **(**Table 4**, Model 1)**.

**Table 4:** Multivariable analysis taking the total FACT-Cog score as an independent variable

**Model 1: Forward linear regression taking the depression score as the dependent variable (without forcing the genetic variables as independent variables)**					
**Variable**	**UB**	**SB**	***p***	**95% Confidence Interval**	
Total FACT-Cog	−0.06	−0.32	< 0.001	−0.10	− 0.03
Sleep quality (PSQI score)	0.30	0.30	0.001	0.13	0.47
Fosaprepitant intake (yes vs no*)	−1.82	−0.19	0.026	−3.43	−0.22
*Reference group; Variables entered in Model 1: carboplatin, total Fact-Cog score, ISI score, PSQI score, fosaprepitant, *OPRM1*.					
**Model 2: Forward linear regression taking the depression score as the dependent variable (while forcing the genetic variables as independent variables).**					
**Variable**	**UB**	**SB**	***p***	**95% Confidence Interval**	
Total FACT-Cog	−0.07	−0.33	0.001	−0.10	−0.03
Sleep quality (PSQI score)	0.33	0.32	0.001	0.14	0.52
Marital status (married vs single*)	−2.53	−0.21	0.029	−4.80	−0.27
Fosaprepitant intake (yes vs no*)	−2.15	−0.22	0.019	−3.94	−0.37
*PER2* (AA vs GG*)	4.60	0.19	0.049	0.02	9.18
*Reference group; Variables entered in Model 2: Marital status, carboplatin, total Fact-Cog score, ISI score, PSQI score, fosaprepitant, *CLOCK*, *PER2*, *CRY2, OPRM1*, *ABCB1*, *COMT*, *DRD2*.					
**Model 3: Forward linear regression taking the anxiety score as the dependent variable (without forcing the genetic variables as independent variables).**					
**Variable**	**UB**	**SB**	***p***	**95% Confidence Interval**	
Total FACT-Cog score	−0.07	−0.32	< 0.001	−0.11	− 0.03
Insomnia severity (ISI score)	0.19	0.26	0.003	0.07	0.32
Fosaprepitant intake (yes vs no*)	−2.17	−0.19	0.022	−4.02	−0.31
*COMT* Met/Met vs (Val/Val + Val/Met)*	1.09	0.17	0.042	0.04	2.14
*Reference group; Variables entered in Model 3: Carboplatin, cyclophosphamide, total Fact-Cog score, ISI score, PSQI score, fosaprepitant, *OPRM1*, *COMT* (Met/Met vs Val/Val + Val/Met).					

**Abbreviations:** UB=Unstandardized Beta and SB=Standardized Beta

When forcing all genetic variables in the model (since our objective was to evaluate genetic factors in particular), the results of a forward linear regression, taking the depression score as the dependent variable, showed that higher total FACT-Cog (B = -0.07), being married compared to single (B = -2.53) and the intake of fosaprepitant compared to not (B = -2.15) were significantly associated with lower depression scores, whereas higher PSQI scores (worse sleep quality) (B = 0.33) and having the *PER2* AA genotype compared to GG (B = 4.60) were significantly associated with higher depression scores **(**Table 4**, Model 2)**.

The results of a linear regression, taking the anxiety score as the dependent variable, showed that higher total FACT-Cog score (B = -0.07) and the intake of fosaprepitant compared to not (B = -2.17) were significantly associated with lower anxiety scores, whereas more severe insomnia (higher ISI scores) (B = 0.19) and having the *COMT* Met/Met genotype compared to those having at least one Val Allele (Val/Val and Val/Met) (B = 1.09) were significantly associated with higher anxiety scores (Table 4**, Model 3**).

## Discussion

Anxiety and depression coexisting during cancer diagnosis and treatment are a source of major distress in breast cancer patients and have a negative impact on the prognosis, treatment adherence, survival rate and quality of life [48]. We conducted this study to explore the prevalence of these two mood disorders in our patients and showed that almost half of the patients self-reported high levels of depression or anxiety (56.2% felt anxious and 43.4% exhibited depressive symptoms). These scores are similar to what was previously reported in Lebanese cancer patients and other breast cancer patients [49, 50].

We then aimed at assessing the association between these symptoms and clinical/genetic factors that have never been previously explored in breast cancer patients (expect for *COMT* rs4680) and we highlighted some new interesting findings. Hence, our results showed that the rs934945 of the *PER2* circadian gene was significantly associated with depression scores, whereas anxiety level was associated with *COMT* rs4680.

To the best of our knowledge, this is the first study to demonstrate that patients carrying the *PER2* AA variant genotype have higher depression scores than patients with GG wild-type genotype. *PER2* is one of the core genes of transcriptional-translational negative feedback loops controlling circadian rhythms and it is expressed in a circadian pattern in the suprachiasmatic nucleus. The rs934945 (G>A) of *PER2* is a coding-region polymorphism with a missense effect, leading to an amino acid substitution from a glycine into a glutamic acid (Gly/Glu) in the *PER2* encoded protein [51]. Some authors identified that the studied SNP is also a CRY-binding domain, and therefore affects the regulation of the circadian rhythm [52]. Several studies reported the importance of the circadian clock mechanisms in neurological pathways regulating affective behaviors including depression [53, 54]. From a monoaminergic point of view, studies in mice showed that PER2 acts as a positive factor in the regulation of monoamine oxidase A gene expression (*MaoA*) in the ventral tegmental area (VTA) and the striatum [53, 54], which could potentially affect the metabolism of serotonin and neurobiology of depression. Nevertheless, more studies are needed to explore the exact molecular effect of this SNP on *PER2* transcription or protein expression/functionality and consequently the impact on the pathogenesis of depression in patients.

Moreover, our study has shown that patients with Met/Met variant genotype for *COMT* p.Val158Met have higher anxiety scores than patients carrying at least one Val allele (Val/Val and Val/Met) as previously described in breast cancer patients [55]. The studied SNP reduces by 3- to 4-fold the enzymatic activity of COMT [56], causing an increased catecholamine levels in patients. Yet, catecholamines are key hormones for stress response and adaptation to the environment; therefore, this increase would lead to stress-induced phenotypes, such as anxiety disorder [57].

The significant associations obtained for *OPRM1* rs1799971, *CLOCK* rs1801260 and depression in the bivariate analyses did not remain in the multivariable analyses. These associations were not previously explored in cancer patients; however, these results are consistent with those reported in other populations that failed to identify any link between the studied SNPs and depression [58–61].

Other than genetic factors, the assessment of clinical factors established that patients who were prescribed fosaprepitant during the intravenous chemotherapy cycle have lower depression and anxiety scores compared to those who did not take it. In fact, chemotherapy-induced nausea and vomiting (CINV) is an irritating common side effect associated with cancer patients [62] causing dehydration, impairment of daily performance and quality of life and electrolyte imbalance in the absence of an adequate prevention and management. The substance P (SP) binds to NK-1 receptors in brain regions involved in vomiting including the brainstem nucleus tractus solitarius and the area postrema but also in the abdominal vagus causing gut contractions [63]. Therefore, fosaprepitant blocks the activity of the SP at the NK-1 receptors and it is indicated for the prevention of acute and delayed nausea and vomiting for patients undergoing highly and moderately emetogenic chemotherapy regimens [64]. Furthermore, evidence suggest that the SP/NK-1 system can also regulate the endocrine system and act as a neuromodulator contributing to brain homeostasis and the sensory neuronal transmission associated with depression, stress and anxiety [63, 65]: the upregulation of the aforementioned system can contribute to the development of depression. Hence, by blocking these receptors, fosaprepitant can have an antidepressant mechanism, which is consistent to what we have found in our study. We have also shown that married women reported less depressive symptoms than single ones which can be explained by the family and social support/empathy they get from their partners, children and friends [66].

Finally, our study identified higher anxiety and depression levels in breast cancer patients suffering from cognitive impairments and worse sleep quality/insomnia. These results have been previously reported in several studies and some authors even suggested that the symptoms seem to be interrelated to form a “cluster of symptoms”: anxiety, depression, sleep disorders and cognitive impairment co-occur and the relationships appear to be bidirectional [7, 67–69]. In that context, reports hypothesized that a higher level of perceived stress and negative mood would lead to acute insomnia and consequently precipitate depression; on the other hand, chronodisruption in patients with chronic insomnia may lead to the first onset of depression. Furthermore, patients with cognitive difficulties might fail to fulfill their social/family obligations and perform their routine activity therefore reaching a lifestyle regularity: all these alteration in circadian rhythm would definitely underpin susceptibility to anxiety, depression and lower sleep quality [70, 71]. The inflammatory/cytokine-based neuroimmunological hypothesis have been presumed to be the common underlying mechanism [72–75]. Therefore, it would be interesting to initiate physical and psychological interventions as of the first chemotherapy session to alleviate emotional distress and all associated symptoms, thus enhancing the overall quality of life.

### Limitations and strengths

Our findings should be interpretated in light of several limitations. Hence, an information and reporting bias could be noted since all clinical measure (anxiety, depression, sleep disorders, cognitive impairment, pain) relied on self-reporting scales. Therefore, patients could have overrated or underrated their symptoms. Moreover, patients might have been exhibiting these mood disorders even before their cancer diagnosis. A selection bias is present since patients were recruited from one hospital only. A residual confounding bias is also possible since not all factors associated with depression and anxiety were considered in this study. Finally, the sample size might be considered as relatively small for genetic analyses and we are aware that a Bonferroni correction should have been applied; nevertheless, to the best of our knowledge, this is the first study to explore a large number of clinical and genetic factors, including genes for the circadian rhythm and stress regulation systems, that have not been previously explored in breast cancer patients, and we performed multivariable analyses to reduce the risk of confounding factors. The Bonferroni adjustment has been considered too stringent and even problematic [76], while a multivariable analysis using the appropriate models allows researchers to adjust multiple testing issues [77]. Thus, we did not make any correction for multiple comparisons during the bivariate analysis; however, all variables that showed a *p* < 0.2 in the bivariate analysis were taken as independent variables in the multivariable analysis; the results of the multivariable analysis were adjusted over the variables that showed a p < 0.2 [78]. In all cases, although our results should be interpreted with caution, they remain interesting since they generate hypotheses for future studies [79]. Further research, including a larger sample of other race/ethnic groups, is needed to confirm our findings and explore their potential translation into clinical practice.

## Conclusion

Our study demonstrated significant associations between cognitive impairment, sleep quality, marital status fosaprepitant intake, and *PER2* polymorphism and depression levels, whereas anxiety levels were significantly associated to cognitive impairment, insomnia severity, fosaprepitant intake, and *COMT* polymorphism. Based on these results, it could be interesting to explore these genetic and clinical factors and identify breast cancer patients with higher risk of exhibiting anxiety and depression. This personalized strategy would help clinicians implement an individualized management therapy aiming at preventing and alleviating the burden of anxiety and depression in patients, thus enhancing the overall quality of life of patients and their families.

## Data Availability

The datasets generated and/or analysed during the current study are not publicly available due to the fact that the study is still ongoing on other cancer populations (other than breast cancer), but are available from the corresponding author on reasonable request.

## Abbreviations

BBB: Blood Brain Barrier
BMI: Body Mass Index
BSA: Body Surface Area
*CLOCK*: Circadian Locomotor Output Cycles Kaput gene
CogPCA: Perceived Cognitive Abilities subscale
CogPCI: Perceived Cognitive Impairments subscale
CogOth: Comments from Others subscale
CogQOL: Impact of perceived cognitive impairments on Quality Of Life subscale
COMT: Catechol-O-methyl transferase
*CRY2*: Cryptochrome circadian regulator 2 gene
*DRD2*: Dopamine receptor 2 gene
EORTC-QLQ C30 scale: European Organization for Research and Treatment of Cancer Quality of Life Questionnaire
FACIT: Functional Assessment of Chronic Illness Therapy system of Quality of Life questionnaires
FACT-Cog: Functional Assessment of Cancer Therapy-Cognitive Function
HADS-A: Hospital Anxiety and Depression Scale; anxiety subscale
HADS-D: Hospital Anxiety and Depression Scale; depression subscale
HPA: Hypothalamic-pituitary-adrenocortical axis
HDF: Hôtel-Dieu de France
ISI: Insomnia Severity Index
PSQI: Pittsburgh Sleep Quality Index
*PER2*: Period 2 gene
P-gp: P-glycoprotein
SD: Standard Deviation
SNPs: Single nucleotide polymorphisms
VAS: Visual Analogue Scale
